# Application of *Wox2a* in transformation of recalcitrant maize genotypes

**DOI:** 10.1007/s42994-023-00116-6

**Published:** 2023-10-13

**Authors:** Qiangbo Liu, Xian Sheng Zhang, Ying Hua Su

**Affiliations:** National Key Laboratory of Wheat Improvement, College of Life Sciences, Shandong Agricultural University, Tai’an, 271018 China

**Keywords:** Genetic transformation, Maize, *Wox2a*, Recalcitrant maize genotypes

## Abstract

The genetic transformation plays an important role in plant gene functional analysis and its genetic improvement. However, only a limited number of maize germplasms can be routinely transformed. The maize gene *Wuschel-like homeobox protein 2a* (*Wox2a*) was shown to play a crucial role in promoting the formation of embryonic cells and enhancing the efficiency of genetic transformation in maize. This commentary discusses the mechanism by which the *Wox2a* gene contributes to the variation in embryogenic tissue culture response among different maize inbred lines. In addition, the frequency and intensity of *Wox2a* or *Wus2*/*Bbm* vector-induced somatic embryogenesis was also discussed. The application of *Wox2a* in transformation of recalcitrant maize genotypes could well accelerate the development of maize genetic improvement.

Plant gene functional analysis and its genetic improvement rely mainly on genetic transformation. Maize is a critical cereal crop in global agriculture, alongside other essential crops like rice, wheat, and barley. Unfortunately, as with these crops, only a limited number of maize germplasms can be routinely transformed, thereby significantly restricting exploration of gene functions and their potential for genetic improvement. Elevating the expression levels of the maize (Zea mays) *Baby Boom* (*Bbm*) and *Wuschel2* (*Wus2*) genes resulted in a significant increase in transformation frequencies in previously non-transformable maize inbred lines. However, some negative effects, including phenotypic abnormality and sterility, were observed in these *Bbm* and *Wus2* overexpression lines. To obtain normal phenotypes for transgenic plants, the desiccation-inducible promoter, *Rab17pro*, was employed to control expression of the *CRE* recombinase gene. This approach successfully eliminated the undesired effects of *Bbm* and *Wus2* overexpression (Lowe et al. 2016). Furthermore, to generate healthy and fertile plants, without any callus formation, Lowe et al. (2018) utilized two other promoters: the maize phospholipid transferase gene promoter (*Zm-PLTPpro*) and the auxin-inducible promoter (*Zm-Axig1*), to drive the expression of *Bbm* and *Wus2*, respectively (Table 1). 

In a recent report by McFarland et al. (2023), the maize gene *Wuschel-like homeobox protein 2a* (*Wox2a*) was shown to play a crucial role in promoting the formation of embryonic cells and enhancing the efficiency of genetic transformation in maize. The T_0_ plants were successfully obtained through the regeneration of somatic embryos derived from the explants transformed with the *Wox2a*-overexpressing vectors. These regenerated plants exhibited a normal phenotype and displayed full fertility (Table 1), suggesting that the *Wox2a* gene has significant potential for applications in the genetic transformation of maize.

**Table 1:** Use of *Bbm*, *Wus* and *Wox2a* to enhance transformation of maize

Vector	Transformation frequencies	Phenotype of regenerated plants	Reference
*Ubi*_*pro*_*::Bbm* or *Nos*_*pro*_*::Wus2*	Low	Stunted, twisted, and usually sterile at maturity	Lowe et al. 2016
*Ubi*_*pro*_*::Bbm* plus *Nos*_*pro*_*::Wus2*	High		
*Rab17*_*pro*_*::moCRE* plus *Ubi*_*pro*_*::Bbm* plus *Nos*_*pro*_*::Wus2*	High	Normal and fertile	
*Zm-PLTPpro::Bbm* plus *Zm-Axig1pro::Wus2*	High	Normal and fertile plants without a callus phase	Lowe et al. 2018
*ZmUbi1::Wox2a*	High	Normal and fertile	McFarland et al. 2023

How was the *Wox2a* gene identified? McFarland et al. (2023) selected *Wox2a* as the candidate gene based on a set of criteria. The *Wox2a* gene was located within the 3 Mb fine-mapping region on chromosome 3, which had a known association with embryogenic culture response, as determined by its genetic position and expression pattern (Salvo et al. 2018). Moreover, in the immature zygotic embryos of A188, a preferred inbred line for tissue culture and transformation applications, the *Wox2a* transcripts exhibited an eightfold increase at 24 h after being introduced into tissues (Salvo et al. 2014). In addition, the *Wox2a* gene showed transcriptional activity in both the zygote and the apical domain of the early embryo in maize. Similarly, its orthologous gene, *AtWox2*, displayed activity in the zygote and played a crucial role in initiating the embryonic shoot apical meristem in *Arabidopsis* (Haecker et al. 2004; Nardmann et al. 2007; Zhang et al. 2017). This suggests that the function of *Wox2a* in embryo development is conserved between maize and *Arabidopsis*.

What is the mechanism by which the *Wox2a* gene contributes to the variation in embryogenic tissue culture response among different maize inbred lines? One assumption suggested that there could be differences in the sequences, or functions, of the *Wox2a* gene between recalcitrant and readily transformable maize genotypes B73 (an essential inbred line in maize genetics and genomics study) and A188. McFarland et al. (2023) determined that the B73 and A188 alleles of *Wox2a* shared only approx. 69% identity in their promoter sequences, whereas the coding sequences for both genotypes displayed a high degree of conservation.

The transgenic cells, transformed with both expression vectors *ZmUbi1::Wox2a-B73* and *ZmUbi1::Wox2a-A188*, showed similar frequencies of somatic embryo formation and plantlet regeneration, indicating that the *Wox2a* gene in different maize inbred lines (B73 and A188), with varying abilities to form embryonic cells, has the same function in embryogenesis and cell differentiation. According to these findings, an alternative assumption proposes that the diverse genetic transformation capacities of B73 and A188 could be attributed to variations in the expression of the *Wox2a* gene. McFarland et al. (2023) detected the *Wox2a* gene expression in maize inbred lines exhibiting varying genetic transformation abilities. They observed that the expression level of *Wox2a* in the highly embryogenic and regenerable maize genotype Hi-II AxB immature embryos was 1.3-fold higher than that in the B73 embryos. This suggests that elevating the expression of the *Wox2a* gene, in the recalcitrant maize genotype, may be sufficient to trigger somatic embryogenesis in its immature embryos.

Hence, the *Wox2a* gene was introduced into the immature zygotic embryos of the maize inbred line LH244, which typically exhibited a slower-growing type I embryogenic callus response, compared to the fast-growing type II response observed in A188. The introduction of the overexpressed *Wox2a* gene resulted in direct somatic embryogenesis in the immature embryos of LH244. Here, some 20–22% of explants treated with *Wox2a* expression, under the *ZmUbi1* promoter, generated somatic embryos at a level significantly higher than the control. However, the *Wox2a* overexpression vector induced somatic embryogenesis at a lower frequency and intensity than *Wus2/Bbm* (Fig. 1 A and B).

**Fig. 1:**
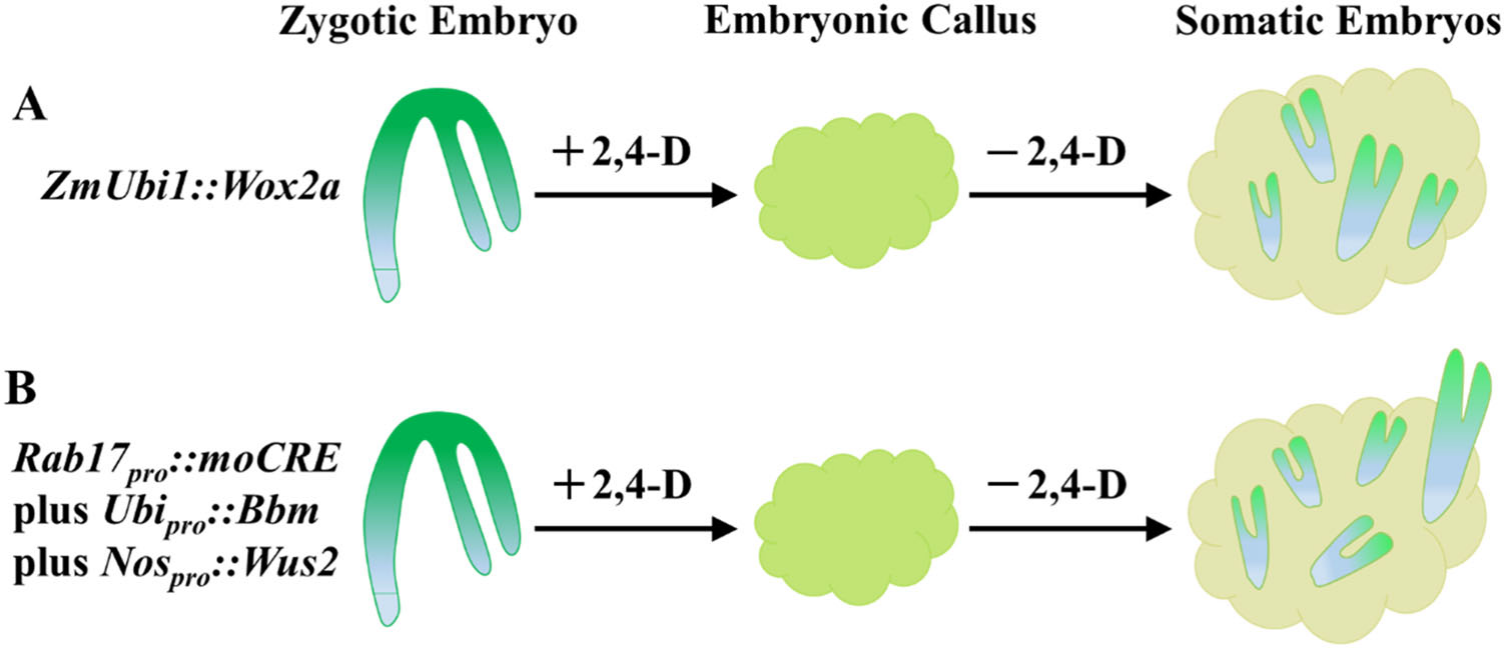
The frequency and intensity of *Wox2a* or *Wus2/Bbm* vector-induced somatic embryogenesis. Maize explants (zygotic embryos) treated with *ZmUbi::Wox2a* (**A**) or *Rab17*_*pro*_*::moCRE/Ubi*_*pro*_*::Bbm/Nos*_*pro*_*::Wus2* (**B**) could generate somatic embryos with a callus phase. The *Wox2a* overexpression vector was significantly simplified relative to the *Wus2/Bbm* vectors. However, the *Wox2a*-based transformation vector induced somatic embryogenesis at a lower frequency and intensity than *Wus2/Bbm*

In summary, McFarland et al. (2023) identified a crucial gene, *Wox2a*, using a forward genetic strategy and established that it could induce somatic embryo formation and promote the growth of friable, embryogenic, and regenerable callus in the recalcitrant maize genotype B73. Of equal importance, *Wox2a* enabled the regeneration of phenotypically normal plants and their subsequent offspring. Integration of these findings into maize genetic breeding programs could well accelerate the development of elite lines with traits tailored for yield performance under regional-specific conditions.

## Data Availability

Data sharing is not applicable to this article, as no datasets were generated or analyzed during the current study.
